# A Proteomic Approach to Lipo-Chitooligosaccharide and Thuricin 17 Effects on Soybean GerminationUnstressed and Salt Stress

**DOI:** 10.1371/journal.pone.0160660

**Published:** 2016-08-25

**Authors:** Sowmyalakshmi Subramanian, Emily Ricci, Alfred Souleimanov, Donald L. Smith

**Affiliations:** Department of Plant Sciences, Macdonald Campus, McGill University, 21111 Lakeshore Road, Sainte Anne de Bellevue, Quebec, H9X3V9, Canada; University of Manitoba, CANADA

## Abstract

Salt stress is an important abiotic stressor affecting crop growth and productivity. Of the 20 percent of the terrestrial earth’s surface available as agricultural land, 50 percent is estimated by the United Nations Environment Program to be salinized to the level that crops growing on it will be salt-stressed. Increased soil salinity has profound effects on seed germination and germinating seedlings as they are frequently confronted with much higher salinities than vigorously growing plants, because germination usually occurs in surface soils, the site of greatest soluble salt accumulation. The growth of soybean exposed to 40 mM NaCl is negatively affected, while an exposure to 80 mM NaCl is often lethal. When treated with the bacterial signal compounds lipo-chitooligosaccharide (LCO) and thuricin 17 (Th17), soybean seeds (variety Absolute RR) responded positively at salt stress of up to 150 mM NaCl. Shotgun proteomics of unstressed and 100 mM NaCl stressed seeds (48 h) in combination with the LCO and Th17 revealed many known, predicted, hypothetical and unknown proteins. In all, carbon, nitrogen and energy metabolic pathways were affected under both unstressed and salt stressed conditions when treated with signals. PEP carboxylase, Rubisco oxygenase large subunit, pyruvate kinase, and isocitrate lyase were some of the noteworthy proteins enhanced by the signals, along with antioxidant glutathione-S-transferase and other stress related proteins. These findings suggest that the germinating seeds alter their proteome based on bacterial signals and on stress, the specificity of this response plays a crucial role in organ maturation and transition from one stage to another in the plants' life cycle; understanding this response is of fundamental importance in agriculture and, as a result, global food security. The mass spectrometry proteomics data have been deposited to the ProteomeXchange with identifier PXD004106.

## Introduction

Soil salinization, one of the most serious agricultural limitations worldwide, is exacerbated by a number of factors including climate (degree of water deficit), the inherent salt content of the soil, topography, underlying geology and hydrology [1]. It is estimated that approximately 20% of irrigated land, which yields one third of the world’s food, is affected by salinity [2,3]. A significant proportion of recently cultivated agricultural land has become saline because of land clearing or irrigation [4] and the impact of irrigation-related salinity on agricultural productivity has been recognized in many parts of the world [5]. In Canada, dry-land salinity is a significant agronomic problem across the prairies [6] where approximately 1 million ha is affected by moderate to severe topsoil salinity [1]. A government of Alberta report suggests the dominant salts in prairie saline seeps are calcium (Ca), magnesium (Mg), sodium (Na) cations and sulfate (SO_4_) anions, and that the impact of moderate to severe soil salinity [electrical conductivity of a saturated paste extract (ECe) of 8 to 16 dS m^-1^ and ECe > 16 dS m^-1^, respectively] is apparent for almost all crops produced under dry-land agriculture conditions in this region, with yield reductions up to 50% in cereals and oilseeds crops [1].

Sodium chloride (NaCl), is a dominant salt in nature; it reduces the ability of plants to take up water (water deficit effect) and other essential nutrients (ion-excess effect) [4, 7]. Salt stress causes changes in plant growth due to (1) osmotically-induced water stress, (2) specific ion toxicity due to high concentration of sodium and chloride, (3) nutrient ion imbalance, due to high level of Na^+^ and Cl^-^ which reduce the uptake of K^+^, NO^-^, PO_4_^3-^ and (4) increased production of reactive oxygen species which damage macromolecules inside plant tissue, all of which result in plant growth reduction [8]. For instance, salt stress enhances the accumulation of NaCl in chloroplasts of higher plants which affects their growth rate, and is often associated with a decrease in photosynthetic electron transport activities [9]. Additionally, in higher plants, it inhibits photosystem (PS)-II activity [10, 11]. Therefore, the presence of saline soils substantially alters plant metabolic processes [12].

Soybean, an important crop legume grown commercially around the world; it is known for the high oil and protein contents of its seeds [13]. Globally, it is one of the main sources for edible vegetable oil and protein and an important livestock feed. For example, the United States of America produced 83,171,600 t while Canada produced 4,246,300 t of soybean in 2011 [14]. In addition, soybean cultivation improves soil health through its ability to fix atmospheric nitrogen (thereby reducing the need for synthetic nitrogen fertilizers) and its deep root system (allowing for soil carbon sequestration) [13]. However, soybean is a salt-sensitive crop [15], and its production is severely affected by saline soils [3]. Soybean exposed to 40 mM NaCl was strongly and negatively affected, while an exposure to 80 mM NaCl was lethal for cv. Enrei [16]. High salinity results in inhibited seed germination and seedling growth, decreased chlorophyll content, reduced nodulation, decreased biomass accumulation, lowered pod numbers and decreased seed weight, and finally, reduced yields [17,18]. In addition, it was shown that salinity inhibits the expansion and curling of root-hairs and reduced the number of nodules in faba bean [19].

Apart from its nutritive value, the soybean-*Bradyrhizobium* symbiosis is one of the most studied in biological nitrogen fixation. A successful interaction between a legume plant and the appropriate rhizobial bacterium leads to the formation of a new plant organ, the nodule, which is generally formed on plant roots; bacteria reside inside the nodule, in the form of bacteroids, and fix atmospheric dinitrogen into ammonia [20]. Nodule formation is a highly specialized two-step process that requires cross-talk between the bacteria and the host plant, wherein the host plants release signal molecules such as flavonoids and iso-flavonoids, which induce the transcription of bacterial nodulation genes leading to the biosynthesis and secretion of lipo-chitooligosaccharides (LCOs), called Nod factors, which act as bacteria-to-plant signal molecules [21, 22, 23]. Nod factors consist of a chitooligosaccharide backbone of three to five β-1,4-linked N-acetyl-D-glucosamine residues, substituted by a fatty acyl chain of varying lengths and with varying degrees of unsaturation, attached at the non-reducing end [24]. Signal molecules, such as LCOs, have been shown to affect aspects of plant metabolism and enhance growth for a variety of agriculturally important plants. For example, LCO has been shown to induce rapid and transient alkalization of tobacco [25] and tomato cells [26] in suspension cultures. Restoration of cell division and embryo development in temperature-sensitive mutants of carrot [27] was observed. LCO treatment of clover seeds enhanced clover nodulation and growth [24]. Inoculation with rhizobia or application of Nod factors (lipo- chitooligosaccharides, LCOs) causes transient increases in cytosolic calcium concentration in root hairs of legume plants [28], leading to enhanced plant growth. Positive LCO effects have also been reported from our laboratory related to enhanced germination and early plant growth in corn, rice, beet, cotton and mung bean [29] and in *Brassica napus* (cultivar Polo) at optimal temperature and low oil cultivar Topas responded to LCO under low temperature (10°C) as well as optimal temperature (25°C) [30]. The first microarray study in our laboratory used spray of LCO on (nodulating OAC Bayfield and non-nodulating Evans x L66-2470); this showed an increase in guaiacol peroxidase activity, while chitinase and β-1,3-glucanase were unaffected [31]. More recent research in our laboratory, on soybean leaves treated with LCOs and under sub-optimal growth conditions, revealed the up-regulation of over 600 genes, of which the largest group of know genes are related to defense and stress, and transcription factors of genes in this category. The microarray results show that the transcriptome of the leaves is highly responsive to LCO treatment at 48 h post treatment under low temperature stress [32]. The results of microarray analysis suggested the need to investigate more carefully the mechanisms by which microbe-to-plant signals aid plants in accommodating abiotic stress conditions.

Some rhizobacteria produce bacteriocins, which increase their adaptability and competitiveness in their specific ecological niche [33]. Bacteriocins are bacterially produced proteins/peptides that are either bacteriostatic or bacteriocidal against strains related to the producer strain [34], thus, they provide competitive advantage for the producer strain [35] and may enhance nodule occupancy when the producer strain is an appropriate member of the rhizobia [36]. In addition, PGPRs can promote plant growth and development via direct and indirect mechanisms [37]. Antibiotic production is one of the mechanisms that PGPR employ to promote plant growth, thereby playing an important role in the bio-control of plant pathogens [38–40]. For example, *Bacillus thuringiensis* NEB17 is a non-bradyrhizobium endophytic bacterium, a PGPR isolated from soybean root tissue [41] that releases the class IId bacteriocin thuricin 17 (Th17), (3,162 Da) [42,43]. Bacteriocins such as thuricin 17 have been shown to enhance plant growth in a variety of crops. Application of Th17 to leaves (spray) or roots (drench) directly stimulates the growth of both soybean and corn [44]. Furthermore, when applied as a co-inoculant with *Brabyrhizobium japonicum* 532C, *B*. *thuringiensis* NEB17 has been shown to enhance soybean root nodulation and plant growth [45].

Seeds and young seedlings are frequently confronted with much higher salinities than vigorously growing plants, because germination usually occurs in the uppermost soil layers, which is generally the site of highest soluble salt accumulation [46]. Since plant species vary in how well they tolerate salt-affected soils, it would be beneficial to enhance crop salt tolerance, using cost effective strategies, in order to meet rising global food demand. Although studies on the role of PGPRs under unstressed conditions are plentiful, the characterization of the beneficial responses to salt stressed plants are few. We attempted to study the modes of action of LCO and Th17 in germinating soybean seeds under unstressed and salt-stressed conditions using a shotgun proteomics approach. In addition, there is limited information available regarding salt-response genes, and the proteins they code for, in the soybean-*Bradyrhizobium* symbiosis. The objective of this work was to understand plant proteomic responses to LCO and Th17 treatment under unstressed and salt-stressed conditions.

## Materials and Methods

### 1. Plant material

Soybean seeds (*Glycine max* (L.) Merrill, cv. Absolute RR) were procured from BelCan, QC, Canada, and used for all the studies on germination and proteomics reported herein.

### 2. Extraction and purification of Lipo-chitooligosaccharides (LCO)

The extraction and purification of LCOs followed the method of Souleimanov et al. [47]. In brief, *B*. *japonicum* cultures were extracted with 40% HPLC-grade 1-butanol. The culture supernatant was carefully removed and condensed in a low-pressure rotary evaporator system (Yamato RE500, Yamato, USA) at 50°C at a speed of 125 rpm, until dryness, and the dried extract was resuspended in 4 mL of 18% acetonitrile. The resuspended extract was loaded onto a C-18 column (PRESEP^™^ Fisher Scientific, Montreal, Canada) and eluted three times using 10 mL of 30% acetonitrile and finally using, 60% acetonitrile. The Nod factors were further isolated and purified by HPLC (Waters 501 pumps, a Waters 401 detector set at 214 nm and a WISP712 autosampler using a C18 reverse phase column (0.46 X 25 cm, 5 μm)—Vydac, CA, USA; catalogue # 218TP54). Chromatography was conducted for 45 min using a linear gradient of acetonitrile from 18 to 60% as described by Souleimanov et al. [47]. Identification of Nod factors was conducted by comparing the retention time of standard Nod factors from strain 532C (identified by mass spectrometry).

### 3. Extraction of Thuricin 17 (Th17)

*Bacillus thuringiensis* NEB17 was cultured in King’s B medium [48] as previously described [42]. In brief, the culture in King’s B medium was incubated on an orbital shaker for 32 h after which this inoculum was subcultured into 4.0 L flasks containing 2.0 L of medium and allowed to grow for 48 h. Th17 isolation and purification was carried out using High Performance Liquid Chromatography (HPLC) using the procedures of Gray et al. [43]. The collected material was denoted as partially purified Th17 and stored at 4°C.

For all experiments, LCO and Th17 were dissolved in water (Cat no. 95304, HPLC grade, Sigma-Aldrich Co., St. Louis, MO, USA) to produce uniform stock solutions and were diluted to the desired concentrations. In all germination experiments LCO concentrations of 10^-6^ and 10^-8^ M, and Th17 concentrations of 10^-9^ and 10^-11^ M were used. These are the concentrations which were found to be the best in previous plant growth response studies [49,50,44].

### 4. Seed germination and elemental analysis

Uniform medium sized seeds were selected and were placed in Petri dishes (Cat. no. 431760, sterile 100 mm x 15 mm polystyrene Petri dish, Fisher Scientific Co., Whitby, ON, Canada) lined with filter paper (09-795D, Qualitative P8, porosity coarse, Fisher Scientific Co., Pittsburg, PA, USA) and containing salt 0, 100, 125, 150, 175 and 200 mM NaCl. Each of the salt concentrations were combined with 10^-6^ M and 10^-8^ M LCO and 10^-9^ M and 10^-11^ M Th17 in order to determine levels of salt tolerance imparted by these bacterial compounds. The plates were incubated in a germination chamber set at 25°C and 70% RH in darkness. Percentage germination was scored at 24, 30, 36 and 48 h after the experiment was initiated. The germinated seeds were then dried for 2 days in an oven at 60°C and fine powdered in a coffee grinder. Approximately 2–4 mg of sample was weighed in a microbalance (Sartorius Pro11, Sartorius Corporation, NY, USA), wrapped in a tin capsule (D1008, Isomass Scientific Inc, Calgary, Canada) and subjected to elemental analysis using a ThermoQuest Elemental Analyzer (Model no. NC 2500, Thermo Quest CE Instruments from Isomass Scientific Inc., Calgary, Canada) to determine any shifts in the pattern for percentage nitrogen, carbon and nitrogen to carbon ratio during the 48 h germination. In brief, the weighed sample in a tin capsule was placed in the autosampler drum, heated to 1000°C, with a constant flow of helium (carrier gas) enriched with a measured amount of high purity oxygen to achieve a strongly oxidizing environment. The combustion gas mixture was driven through an oxidation catalyst (Cr_2_O_3_) zone to achieve quantitative oxidation and subsequently through a zone of copper to reduce nitrogen oxides formed during combustion and catalyst oxidation, to elemental nitrogen and scrub excess oxygen. The gas mixture (N, CO_2_, H_2_O) was passed through a trap containing anhydrone to adsorb water. The resulting components of the combustion mixture were eluted and separated by a Porapack PQS column and subsequently detected by a TCD in the sequence N, CO_2_ [51].

### 5. Label free proteomics

For the proteome analysis, germinated seeds from the unstressed condition, including the water-only control, 10^-6^ M LCO, 10^-9^ M Th17, and the salt stressed condition, including 100 mM NaCl as the salt-stressed control, and combinations of 100 mM NaCl with 10^-6^ M LCO and 10^-9^ M Th17, were sampled and total proteins extracted using a protein extraction kit (Cat. no. PE-0230, Plant total protein extraction kit, Sigma-Aldrich, Co., St. Louis, MO, USA).

#### 5.1. Protein extraction

In brief, the sampled seeds (pool of 10 seeds per replicate) were ground to a fine powder in liquid nitrogen. Approximately 100 mg of the fine powder was placed in sterile eppendorf tubes and 1 mL of ice cold methanol (Cat no. 15468–7, Sigma-Aldrich Co., St. Louis, MO, USA) was added, vortexed, incubated in -20°C for 20 min. and centrifuged (Micro12, Fisher Scientific, Denver Instrument Co., USA) at 13,000 rpm for 7 min. at 4°C. The supernatant was discarded and the procedure was repeated twice more, followed by similar incubation in acetone (Cat. no. 179124, Sigma-Aldrich, Co., St. Louis, MO, USA), both steps in order to remove phenolics and secondary metabolites that might otherwise interfere with LC-MS/MS analysis. The RW2 solution was added to the samples after removing acetone, vortexed for 30s and incubated at room temperature (22°C) for 15 min. The samples were then centrifuged at 13,000 rpm for 10 min and the supernatant carefully collected in a fresh sterile tubes. The supernatant constituted total proteins from that sample. The proteins were then diluted and quantified using the Lowry method, and samples of 10 μg in 20 μL of 1M urea were taken to the Institut de recherches cliniques de Montréal (IRCM) for label free proteomic analysis using LC-MS/MS.

#### 5.2. Proteome profiling

The total proteins extracted were then digested with trypsin and subjected to LC-MS/MS using a Velos Orbitrap instrument (Thermo Fisher, MA, USA). Tandem mass spectra were extracted, charge state deconvoluted and deisotoped and all MS/MS samples were analyzed using Mascot (Matrix Science, London, UK; version 2.3.02). Mascot was set up to search the Soybean_20120801 database (unknown version, 80416 entries) assuming the digestion enzyme trypsin. Mascot searched with a fragment ion mass tolerance of 0.60 Da and a parent ion tolerance of 15 PPM. Carbamidomethyl of cysteine was specified in Mascot as a fixed modification. Oxidation of methionine was specified in Mascot as a variable modification.

#### 5.3. Criteria for protein identification

Scaffold (version Scaffold 4), Proteome Software Inc., Portland, OR), was used to validate MS/MS based peptide and protein identifications. Peptide identifications were accepted if they could be established at greater than 95.0% probability, as specified by the Peptide Prophet algorithm [52]. Protein identifications were accepted if they could be established at greater than 99.0% probability and contained at least 2 identified peptides. Protein probabilities were assigned by the Protein Prophet algorithm [53]. Proteins that contained similar peptides, and could not be differentiated based on MS/MS analysis alone, were grouped to satisfy the principle of parsimony.

### 6. Data analysis

Experiments were structured following a completely randomized design. The SAS statistical package 9.4 (SAS Institute Inc., Cary, NC, USA.) was utilized. The Proc Mixed procedure and Tukey’s multiple means comparison were used to determine differences among means at the 95% confidence level for the germination and elemental analysis data.

Scaffold 4 was used to analyze the proteomics data for fold change and Fisher’s exact test of the identified proteins, after subjecting the quantitative value of the spectra to the embedded normalization. The FASTA file generated was analyzed using Blast2GO-Pro 3.1.3 [54–57], for the functional annotation and analysis of the protein sequences. Apart from these, Enzyme code (EC), KEGG maps and InterPro motifs were queried directly using the InterProScan web service. The mass spectrometry proteomics data have been deposited to the ProteomeXchange Consortium (http://proteomecentral.proteomexchange.org) via the PRIDE partner repository [58] with the dataset identifier PXD004106.

## Results

### 1. Seed germination and elemental analysis

**Fig 1:**
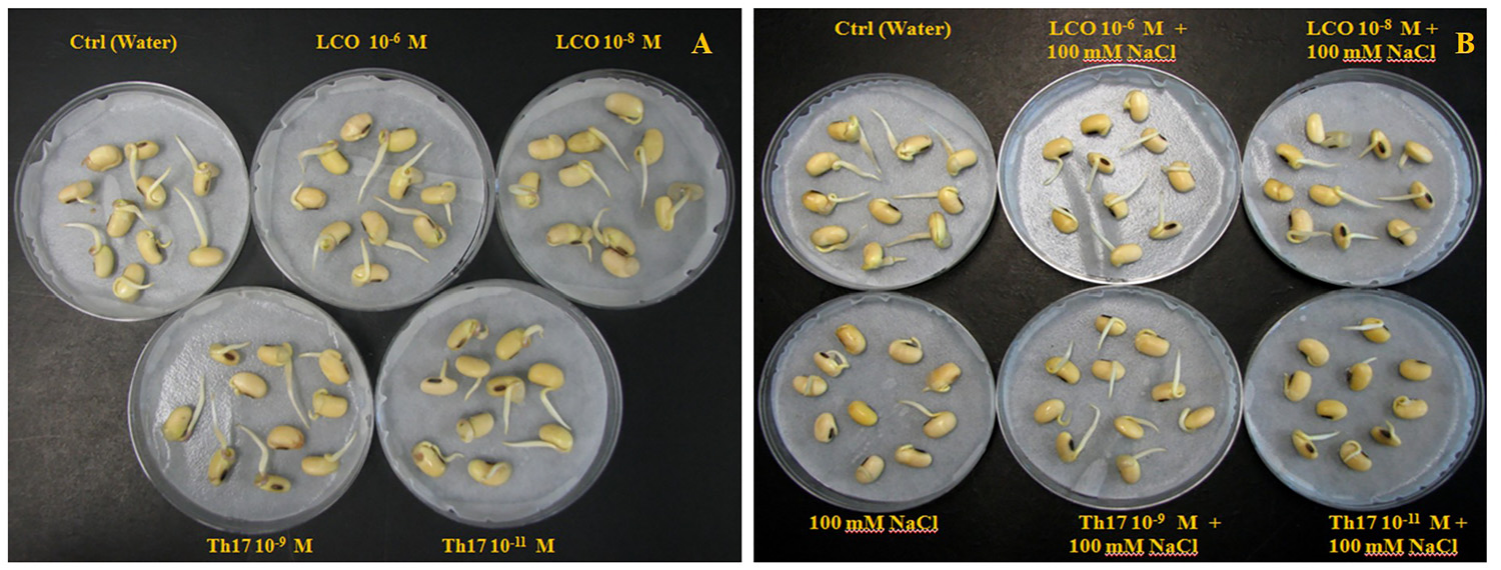
Soybean seed germination 48 h post treatment (un-stressed—Panel A; 100 mM NaCl stress—Panel B).

**Fig 2:**
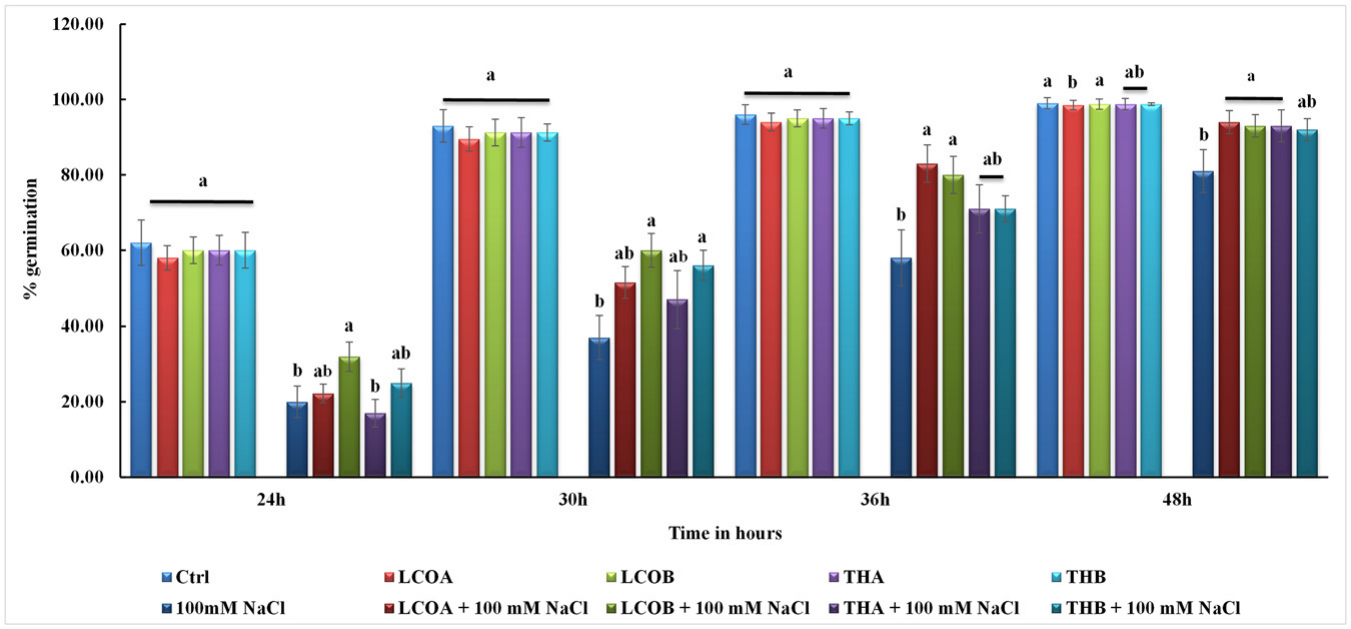
Bar chart representing soybean seed germination under optimal and 100 mM NaCl stress.

Bacterial signal compounds LCO and Th17 on soybean seed germination was evaluated in this study under optimal and salt stress conditions. Germination pattern was studied on seeds treated using two concentrations of LCO (10^-6^ and 10^-8^ M) and Th17 (10^-9^ and 10^-11^ M) in combination with 100, 125, 150, 175 and 200 mM NaCl. Salinity stress severely affected seed germination by delaying the onset of germination in this cultivar with increase in salinity stress levels (S1 Table for germination data on all NaCl concentrations tested). LCO 10^-6^ M and Th17 10^-9^ M helped germinating soybean seeds overcome salinity stress and did this most effectively at 100 mM NaCl (Figs 1 and 2), when measured at 48 h after the onset of germination, although seeds continued to germinated at higher levels of stress. However, in the absence of stress, the compounds did not result in statistically significant differences in the germination pattern.

**Fig 3:**
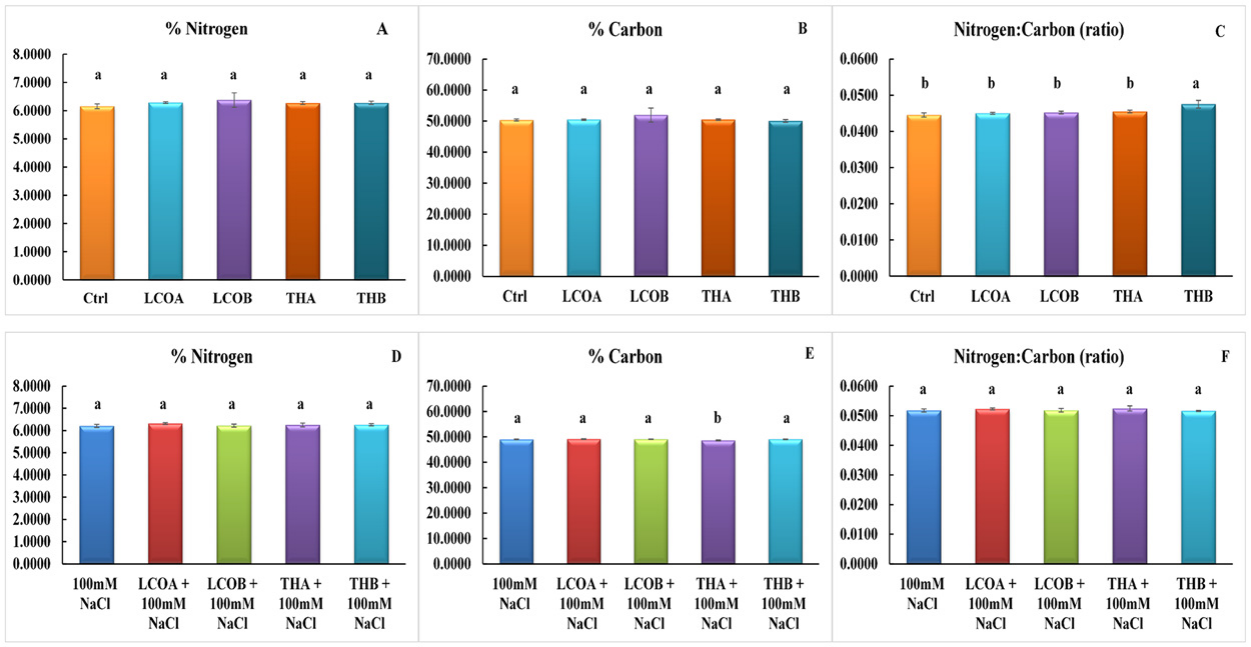
Elemental analysis of soybean seeds after 48 h from the onset of germination. Panels A, B, C represent data for unstressed treatments and Panels D, E, F represent data for stressed treatments.

In order to understand in part the above effect, elemental analysis for nitrogen and carbon percentages in the 48 h germinated seeds was conducted. Germinated seeds under unstressed and 100 mM NaCl and in the presence of signals did not show any statistically significant differences in the percentage of nitrogen and carbon, suggesting that the nitrogen and carbon percentages in the 48 h germinated seeds were very similar in the two groups (Fig 3). However, as the salt stress levels increased, subtle effects on the nitrogen and carbon percentages and the N:C ratio was evident. (S2 Table for elemental analyses data for all the above mentioned salt stress levels). Hence a label free proteomic approach was considered in order to study the effects of the signal compounds and salt stress on germination, 48 h germinated seeds from the unstressed (treatments include Control, 10^-6^ M LCO and 10^-9^ M Th17) and salt stressed (treatments include 100 mM NaCl Control, 10^-6^ M LCO + 100 mM NaCl and 10^-9^ M Th17 + 100 mM NaCl). (Please note—for all proteomics work, the treatments will be referred to as control, LCO, Th17, 100 mM NaCl control, LCO + 100 mM NaCl and Th17 + 100 mM NaCl).

### 2. Proteome profiling

To understand the effect of LCO and Th17 on unstressed and salt stressed germinated seeds, total proteins were extracted from the samples and subjected to LC-MS based proteome profiling. Based on the quantitative value of the identified spectra, and at 99% protein probability, with 2 minimum peptides and 95% peptide probability the following number of proteins were identified (Table 1).

**Table 1:** Total number of proteins identified at 99% protein probability and total spectra at 95% peptide probability, with 2 minimum peptides.

Unstressed	Control 1	Control 2	Control 3	LCO 1	LCO 2	LCO 3	Th17 1	Th17 2	Th17 3
**Proteins**	243	282	217	272	263	228	291	266	226
**Spectra**	6465	4901	5864	4513	4442	5333	4748	4622	4781
**Stressed at 100 mM NaCl**									
**Proteins**	228	231	219	228	257	215	262	210	204
**Spectra**	4263	4847	5850	4339	4662	6150	4834	4211	4651

The treatment contrasts were then analyzed for fold-change after normalization, and Fisher’s Exact test was used to narrow down the set of up- and down-regulated proteins, to predict their probable functions at the 48 h time point. It is likely that we missed some of the proteins due to very strict criteria for difference detection during data analysis; however, this level of stringency was utilized for ease of subsequent functional interpretation. According to the fold-change patterns and Fisher’s Exact test of the contrasts, the proteins were categorized as known proteins, predicted proteins, unknown proteins and unnamed protein products (S1 and S2 Data).

Based on the known and predicted proteins, some of the prominent proteins in unstressed control seeds included metallothionein, stearoyl-acyl carrier, thioredoxin, phosphogluconate dehydrogenase, a 97 kDa heat shock protein (HSP), α and β-subunits of conglycinin, glycinin, lipoxygenase 1, 2 and 3, embryonic protein DC8 like and sucrose binding protein. Glutathione S transferase, peroxisomal voltage dependent anionic channel (VDAC) protein, PEP carboxylase, uricase, alcohol dehydrogenase, arginosuccinate synthase, phosphoglycerate kinase, importin subunit, IN2 homologue, oleosin isoform, and universal stress protein were some of the notable proteins up-regulated by LCO treatment. The Th17 treated seeds were up-regulated for the proteins mentioned for LCO treatment, and also showed a marked increases (2-fold and above) in auxin-like protein, Rubisco oxygenase large subunit, Kunitz type trypsin inhibitor, stearoyl acyl carrier, isocitrate lyase and pyruvate kinase. Both LCO and Th17 caused up-regulation of PEP carboxylase but the α- and β-subunits of conglycinin, glycinin showed a marked down-regulation as compared to control (S1 and S2 Data) for Fold change and Fisher’s Exact test results for soybean signals treatment contrasts).

The number of significant proteins identified in the salt stressed treatments was remarkably lower than the signals-only group, especially in the Control vs LCO and Control vs Th17 contrasts. The salt stress control (salt stress in the absence of signals) had increased levels of aspartic proteinase, PEP carboxylase, lipoxygenase 1, predicted dehydrin-like protein and mannose phosphate isomerise, while the salt stress with LCO caused up-regulation of glutathione-S- transferase, a predicted auxin-induced protein, pyruvate kinase, importin subunit, and predicted PR5 protein. The salt stress with Th17 treatment caused up-regulation of aspartic proteinase, predicted auxin-induced protein, mannose phosphate isomerase, glyceraldehydes phosphate dehydrogenase, Lea protein, Bowman-Birk proteinase inhibitor, oleosin isoform A and B and a dehydrin-like protein, as compared to salt stress alone (control for this group) and LCO.

**Fig 4:**
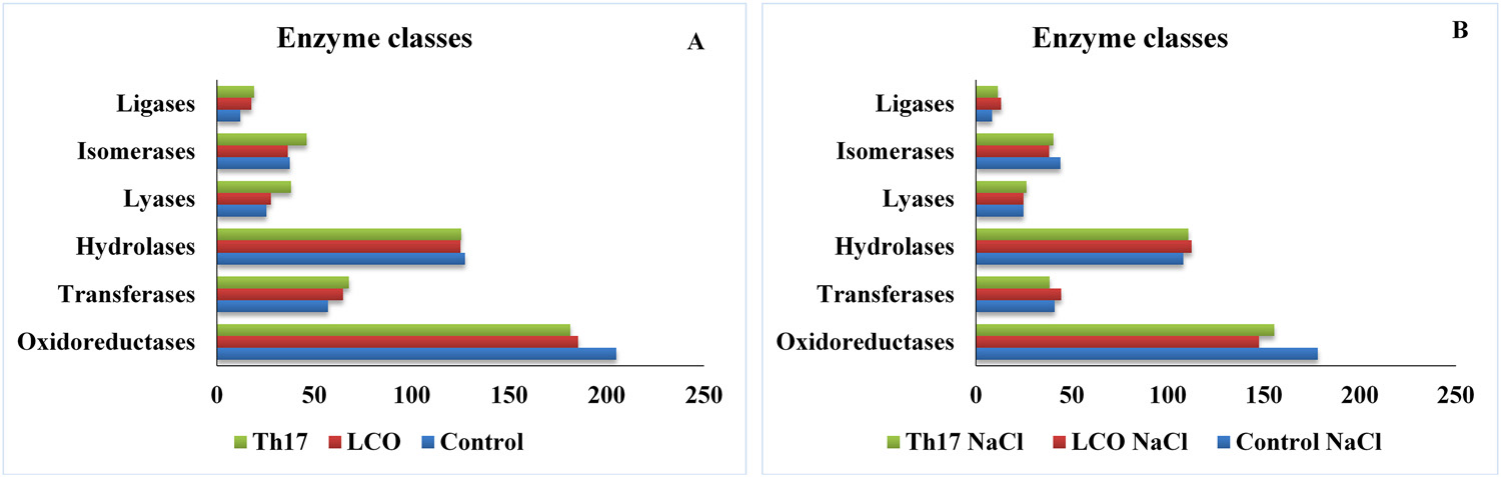
Enzyme code distribution categorized based on main enzyme classes in unstressed and salt stressed treatments.

Based on Blast2GO Pro results, the enzyme code distribution for both the unstressed and salt stressed seeds were studied. A sharp decrease in some of the main enzyme classes was observed in the salt stress group as compared to the unstressed group. Lyases remained unchanged in the control and salt control treatments. An increase in isomerases was observed in the salt control (17.3%) and in LCO with 100 mM NaCl treatment (5.4%) (Fig 4 panel A, B; Table 2).

**Table 2:** Enzyme classes represented in the unstressed and 100 mM NaCl stressed treatments.

EC Classes	Control	LCO	Th17	Control NaCl	LCO NaCl	Th17 NaCl
**Oxidoreductases**	205	185	181	178 (↓ 14.10%)	147 (↓ 22.9%)	155 (↓ 15.48%)
**Transferases**	57	65	68	41 (↓ 32.65%)	44 (↓ 38.53%)	38 (↓ 56.60%)
**Hydrolases**	127	125	125	108 (↓ 16.17%)	112 (↓ 10.97%)	111 (↓ 11.86%)
**Lyases**	25	28	38	25	25 (↓ 11.32%)	26 (↓ 37.5%)
**Isomerases**	37	36	46	44 (↑ 17.3%)	38 (↑ 5.4%)	40 (↓ 13.95%)
**Ligases**	12	18	19	8 (↓ 40%)	13 (↓ 32.25%)	11 (↓ 53.33%)

**Fig 5:**
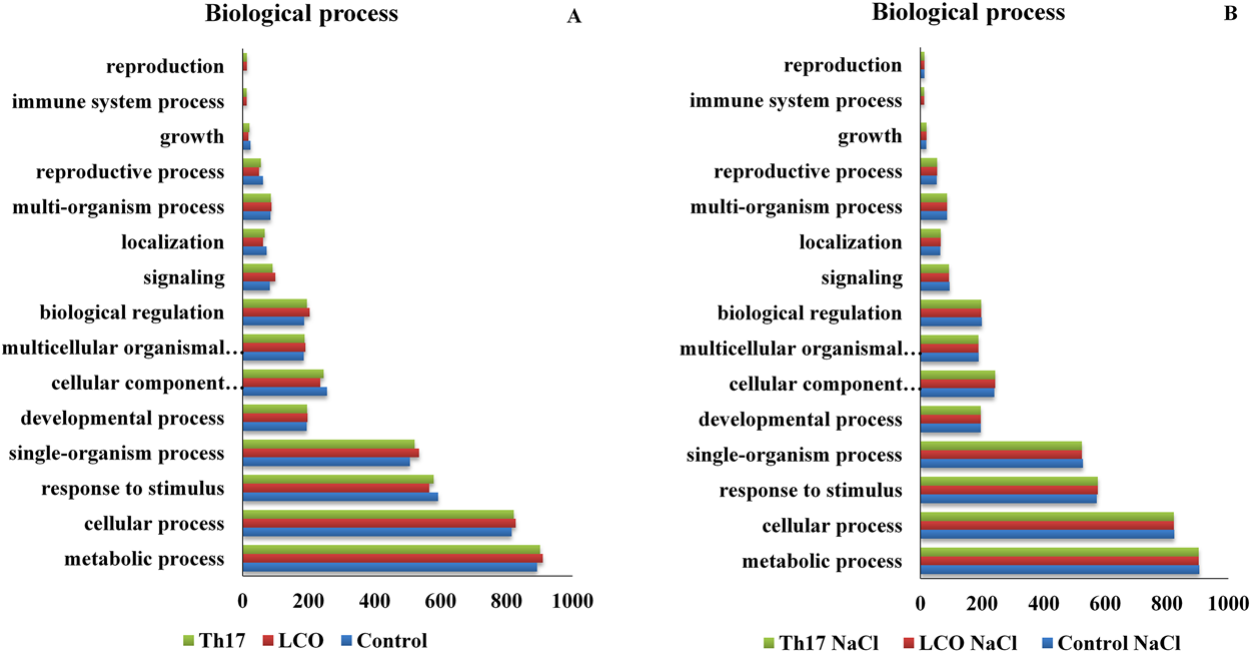
Functional classification of the GO distribution for biological process in unstressed and salt stressed treatments.

**Fig 6:**
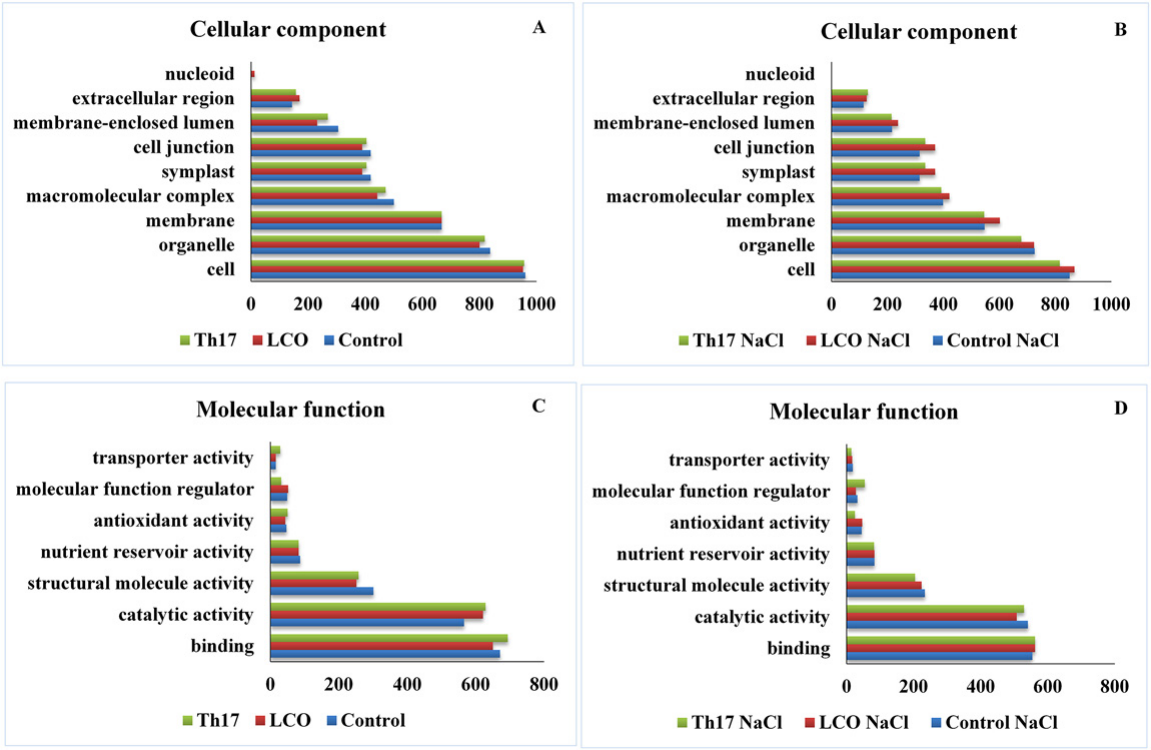
Panel A, B—Functional classification of the GO distribution for cellular components in unstressed and salt stressed treatments; Panel C, D—Functional classification of the GO distribution for molecular function in the unstressed and salt stressed treatments.

The GO function distribution characteristics of the unstressed and salt stressed treatments also indicated that the proteins identified were mostly associated with the carbon metabolism, respiratory electron transport, tricarboxylic acid cycle metabolism, amino acid metabolism, protein import, protein processing, protein assembly, transcription, membrane transport, antioxidant defense, nutrient reservoir proteins, and proteins associated with abiotic stresses such as salt, cold and heat (Fig 5 panel A and B; Fig 6 –panel A, B, C and D). Molecular function, biological processes and cellular components were all affected in both unstressed and salt-stressed conditions. There was a down-regulation of most of the important components in all the above mentioned functions under salt stress, and especially so with ATPase activity, heme binding, oxidation-reduction processes, proteosome core and regulatory complex subunits, photorespiration, meristem structural organization, response to oxidative stress and leaf morphogenesis related proteins. However, other functional classes such as cation binding, nucleotidyltransferase activities, nucleosome and nucleosome assembly, GTP catabolic processes, cytoskeleton organization and mature ribosome assembly proteins were up-regulated in the salt stress group. There was no change in the patterns of GTP binding, protein heterodimerization activity, translation elongation factor activity, protein folding, cytoplasmic and mitochondria proteins (S3 Table).

**Fig 7:**
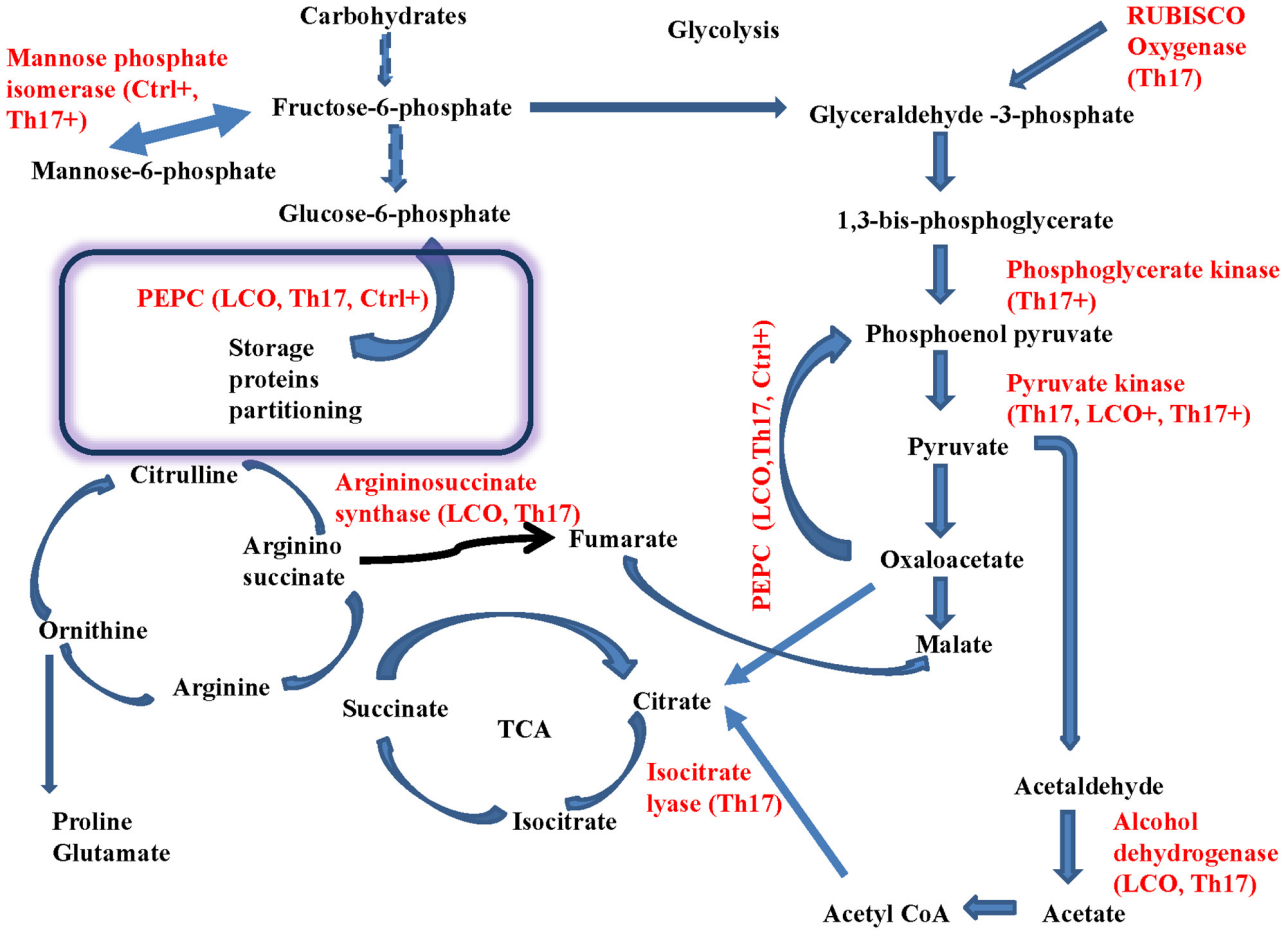
Co-regulation of metabolic pathway components in soybean seeds after 48 h from the onset of germination, as reflected by label free proteomics data. Those in red are the up-regulated proteins in the different treatments mentioned in brackets.

Based on the known and predicted proteins, as identified using fold-change and Fisher’s exact test, we generated a metabolic pathway map (Fig 7) that could explain, at least in part, the reason how and why the signal compounds LCO and Th17 promoted seed germination under unstressed conditions and compensate for negative effects on germination under salt stress.

## Discussion

Seed germination is an important event in the initiation of a plant’s life and hence is a complicated physiological process. This event is of importance in agriculture since under favourable circumstances, many biochemical processes are revoked and new processes are initiated and these dictate the development and establishment of a new seedling. The major LCO molecule produced by *B*. *japonicum* 532C, Nod Bj V (C18:1;MeFuc), isolated and identity confirmed in our laboratory, has been reported to have a positive and direct effects on both legume and non-legume seed germination, and plant growth and development [29]. LCO has been shown to enhance seed germination and seedling establishment for soybean, common bean, maize, rice, canola, apple and grapes, and is usually accompanied by increased photosynthetic rates [59]. Microarray studies of soybean leaves treated with LCOs under sub-optimal growth conditions up-regulated over 600 genes. Many of these were defense and stress response related, or transcription factors related to these responses, suggesting that the effect of LCO at the transcriptome of the leaves at 48 h post treatment are at least partly centred around stress responses [32]. These results suggest the need to further investigate the mechanisms by which microbe-to-plant signals might help plants accommodate abiotic and biotic stress conditions. However, Th17 has not been as well studied as LCO, as it is was more recently isolated, although we have some information regarding its effects on soybean and corn plant growth [37, 44].

Hence, in this study we performed germination tests to evaluate the efficacy of both LCOs and Th17 on germination under unstressed and salt stressed conditions for the soybean cultivar absolute RR. These compounds alleviated salt stress inhibition of germination up to 150 mM NaCl, with the best response seen at 100 mM NaCl. As the concentration of salt increased in the treatments, there was a delay in the onset of germination and resulted in reduced seed germination, corroborating with similar patterns observed in previous studies on soybean germination under salt stress, despite differences in the cultivars studied [60]. The germinated seeds from the treatments at 48 h were subjected to elemental analysis in order to understand the shift in the nitrogen and carbon metabolism during seed germination. Previous studies on assimilate partitioning between storage products in soybean zygotic embryos (from developing seeds) [61–64] and on soybean somatic embryo using the somatic embryo model system based on Soybean Histodifferentiation and Maturation medium (SHaM) under different C:N ratios [65] suggests that changes in the C:N ratio influence the partitioning of storage products especially proteins and oil. During the process of germination these partitioned storage products are broken down to supply for the emerging seedling [66,67]. Since the bacterial signals altered the germination patterns under salt stress, we extended these thoughts into studying the nitrogen and carbon responses towards bacterial signals and accompanied with a stress on germinating seeds. Although the signals and signals in combination with 100 mM NaCl did not show any statistically significant difference, subtle differences started emerging as the salt stress increased. It is probable that these differences increase as the germination progresses beyond the 48 h time point mentioned in this study.

With the advances in instrumentation and bioinformatic analysis, it is now becoming evident that proteins and effects on proteins can provide key evidence regarding shifts in plant physiology, since they play central roles in essentially all metabolic processes. Despite these advances, proteome profiling in systems biology is still a major challenge. However, the amount of information they can add to the understanding of a biological system is impressive. In this study we used the label free proteomics approach to understand soybean seed germination in the presence of microbial signal compounds and under unstressed and 100 mM NaCl stressed conditions.

Metallothioneins are a group of proteins with highly conserved cysteines and have metal binding attributes that are important for plant nutrient acquisition, survival and development [68]. Plant metallothioneins are of at least of four types, 1–4 (pMT); some information is available regarding soybean pMT type 4, the transcripts of which are approximately 375 fold more abundant in seeds than to other soybean seed pMTs [69]. The presence of metallothionein in our study might indicate the up-regulation of molecular functions related to copper, zinc, heme, metal ion binding, and the nutrient reservoir activities. Thioredoxins comprise a group of proteins that modulate redox potential and regulate various enzymatic processes in metabolic pathways, and act as functional components of antioxidant pathways [70,71]. Thioredoxin from soybean nodules has been reported to play an important role in the nodulation process and in nitrogen metabolism [72,73]. During nodulation, nodulin-35, a subunit of uricase is proposed to be a possible target for thioredoxin [73]. The presence of phosphoenol pyruvate carboxylase in our contrasts is not surprising since, the interactions of thioredoxins with the enzymes of carbon metabolism have been demonstrated earlier [74].

The major storage proteins of soybean seeds are α and β-subunits of conglycinin and glycinin. β-conglycinin and glycinin degrade rapidly and their degradation products accumulate or degrade further as the seeds germinate. This degradation, by proteolysis, provides amino acids and energy for the growing seedlings [75]. Both LCO and Th17 caused up-regulation of PEP carboxylase, and degraded the α- and β-subunits of conglycinin, and glycinin as compared to the levels in control treatment seeds. Degradation of seed storage proteins is carried out by thioredoxins in cereals [76], and it is probable that similar mechanisms occur in soybean seeds as well, given the faster rate of storage protein depletion in LCO and Th17 treated seeds. In non-photosynthetic tissues such as oil-containing seeds, phosphoenol pyruvate plays an important role in channelizing carbohydrates to plastidic fatty acid oxidation, production of ATP in the mitochondria, replenishing tricarboxylic acid pathway intermediates and synthesis of organic acids for the biosynthesis of essential amino acids and in nitrogen assimilation. All these coordinate the efficacious use of seed storage proteins during seedling establishment [66,67,77,78]. This is indicative of efficient storage protein utilization in conjuction with thioredoxin and PEP carboxylase, a probable reason why we have often observed better germination patterns at early stages of germination (up to 48 h) for signal compound treated seeds. Lipoxygenase 1, 2 and 3 are essential for storage protein break down and also for the biosynthesis of chloroplasts in soybean seeds [79,80]. Embryonic protein DC8 like, and sucrose binding protein, were the other proteins down-regulated in LCO and Th17 treated seeds as compared to control. It is probable that the lipid metabolism and the glycolytic pathway in the germinating seeds is not directly controlled by these proteins but by an alternate mechanism.

Glutathione-S-transferase and an associated IN2 homologue, peroxisomal voltage dependent anionic channel (VDAC) protein, alcohol dehydrogenase, arginosuccinate synthase, phosphoglycerate kinase, importin subunit, oleosin isoform (an oil body membrane protein that acts as an emulsifier for lipid storage in seeds), LEA and universal stress protein were some of the notable proteins up-regulated in germinating LCO treated seeds. Glutathione-S-transferase and an associated IN2-1 homologue B-like protein are the major antioxidant proteins observed in soybean seeds [3]. Although the function of GST in seeds is not fully understood, its presence during stress responses is indicative of probable oxidative damage protection. Reports that GST has some control over the regulation of the phytohormones GA and ABA [81] is encouraging in that the regulation of these hormones during seed germination by LCO and Th17 could be of significance in agriculture. The fold change increase in alcohol dehydrogenase in LCO and Th17 treated seeds was not a surprise, as the decreased levels of oxygen in germinating seeds favours fermentation, and this in turn could also increase the success of germination [82]. LEA proteins are seen to accumulate during the final stages of seed development [83] and seen to be expressed in soybean seeds during salt stress [84]. Along with the universal stress proteins, LEA proteins probably help seeds adapt for eventual salt stress responses. Voltage dependent anionic channel proteins are recognized as those that are primary transporters of ions and metabolites across organelle membranes, especially so with outer mitochondrial membrane and peroxisomes [85]. Peroxisomes control fatty acid metabolism, energy metabolism (the pentose-phosphate pathway) and glyoxylate metabolism of germinating seeds, allowing conversion of storage lipids into sugars [86]. The up-regulation of peroxisomal voltage dependent anionic channels (pVDAC) in both LCO and Th17 treated seeds suggests increased energy metabolism associated with mitochondrial function, as seen from the GO function outputs.

The Th17 treated seeds manifested the above mentioned proteins, seeds also manifested them as a result of LCO treatment, but LCO treatment also caused a marked increase (2-fold and above) in auxin-like protein, down regulation of rubisco oxygenase large subunit, Kunitz type trypsin inhibitor, stearoyl acyl carrier, isocitrate lyase and pyruvate kinase. Stearoyl acyl carrier up-regulation indicates the regulation of stearic acid contents in the seeds, which is of importance in soybean seed oil composition [87]. Soybean cv. Jefferson has been reported to up-regulate β-conglycinin, glycinin, Kunitz trypsin inhibitor, alcohol dehydrogenase, Gm Bd 28K allergen, seed maturation proteins and sucrose binding proteins in seeds during germination [88,89]. Transgenic soybean seeds have higher amounts of malondialdehyde, ascorbate peroxidase, glutathione reductase, and catalase (29.8, 30.6, 71.4, and 35.3%, respectively) than non-transgenic seeds. Precursors of glycinin, allergen Gly m Bd 28k, actin and sucrose binding proteins were the other proteins identified [90,91].

The number of proteins identified in the salt stressed signals group was remarkably lower than the unstressed signals group, especially in the control vs LCO and control vs Th17 contrasts; this is very typical of soybean seed salt tolerance responses, as found in previous studies of soybean salt stress [92]. Aspartic proteinase has been observed regularly under salt stress, although its function in salt stress is unknown. In the salt-stress control seeds PEP carboxylase, lipoxygenase 1, and mannose phosphate isomerise manifested increased glycolytic processes, while the predicted dehydrin-like protein could be used for compensation related to the water deficit effects of salt stress [93]. Salt stress in the presence of LCO resulted in up-regulation of glutathione-S- transferase, a predicted auxin-induced protein, pyruvate kinase, importin subunit, and predicted PR5 protein. GST and PR5 protein are stress regulators [94,95]. Salt stress in combination with Th17 caused up-regulation of aspartic proteinase, predicted auxin-induced protein, mannose phosphate isomerase, glyceraldehydes phosphate dehydrogenase, Lea proteins, Bowman-Birk proteinase inhibitor, oleosin isoform A and B and a dehydrin-like protein, as compared to salt stress control treatment and salt stress with LCO treatment. Kunitz trypsin inhibitor and Bowman-Birk proteinase inhibitor are the two major trypsin inhibitors of soybean that control the degradation of storage proteins. Along with GST, they help protect seeds from predators [75].

In previous studies of salt stressed soybean seeds, the levels of proteins such as kinesin motor protein, trypsin inhibitor, alcohol dehydrogenase and annexin, were found to change, suggesting that these proteins might play roles in soybean salt tolerance and adaptation [16,96]. The percentage germination was not affected in soybean cultivars Lee68 and N2899 (salt-tolerant and salt-sensitive respectively) when exposed to 100 mM NaCl. The mean germination time for Lee68 (0.3 days) and N2899 (1.0 day) was delayed, compared with control plants. Hormonal responses to salt stress differed between these cultivars. Increased abscisic acid levels and decreased giberrelic acid (GA 1, 3) and isopentyladenosine concentrations was seen in both these cultivars; auxin (IAA) increased in Lee68, but remained unchanged in N2899. Two dimensional gel electrophoresis, followed by MALDI-TOF-MS analysis, also suggested increases in ferritin and the 20S proteasome subunit β-6 in both the cultivars while, glyceraldehyde 3-phosphate dehydrogenase, glutathione *S*-transferase (GST) 9, GST 10, and seed maturation protein PM36 were down-regulated in Lee68. These proteins were present at low concentrations in N2899 in the absence of stress, and were seen to up-regulate following exposure to salt stress [3].

In our study, although strict statistical criteria were used to identify and understand the role of these proteins in unstressed and salt stressed seeds, this combination of up- and down-regulated proteins suggested a metabolic shift and represents a strategy used by soybean seeds to enhance tolerance of, or adaptation to, salt stress in the presence of LCO and Th17. However, relaxation of statistical criteria could reveal the presence of more proteins, and provide broader insights; however, these would be associated with a lesser degree of certainty. Although functional roles for all the known proteins identified by label free proteomics needs future meticulous work, it would be inappropriate to reject the role of the many hypothetical, unknown proteins and the unnamed protein products that were up-regulated in the seeds, as they could also add to our understanding of currently known adaptation strategies.

## Conclusions

In this study, we compared the effects of LCO and Th17 under unstressed and salt-stressed conditions; this is the first study conducted to determine chronic exposure effects of these signals, in combination with stressful levels of salt, on germinating soybean seeds. This subtropical crop requires temperatures of 25–30°C for optimum growth and is severely affected by salinity stress. Although LCO is commercially available (Optimize with LCO promoter technology, and a wide range of similar products) and is known to speedup plant growth in the field, the comparison between LCO and Th17 for seed germination and the effects on the proteome of these seeds under stressed and unstressed conditions enhances its potential for better commercialization. In addition, the use of such growth promoting technologies might help invigorate elements of the native soil microflora and create synergies among them, promoting the potential for decreased use of chemical inputs in the cultivable land, and perhaps enhanced crop productivity on salinized soils around the world.

## Supporting Information

S1 Data Fold change—soybean seed germination at 48 h. (XLSX)

S2 Data Fisher’s test—soybean seed germination at 48 h. (XLSX)

S1 Table Least square means of soybean germination treated with lipo-chito-oligosaccharide and thuricin 17 under optimal and salt stress conditions. (DOCX)

S2 Table Least square means of elemental analysis data on 48 h post germinated soybean (Treatments include lipo-chito-oligosaccharide and thuricin 17 under optimal and salt stress conditions. (DOCX)

S3 Table GO function categories amongst un-stressed and salt stressed groups. (DOCX)
