# Supplementary material for: A Proteomic Approach to Lipo-Chitooligosaccharide and Thuricin 17 Effects on Soybean GerminationUnstressed and Salt Stress
Source: PLoS One. 2016 Aug 25;11(8):e0160660. doi: 10.1371/journal.pone.0160660 (PMC4999219; doi:10.1371/journal.pone.0160660)
Supplement: S1 Table — (DOCX) [file pone.0160660.s003.docx]

S1 Table: Least square means of soybean germination treated with lipo-chito-oligosaccharide and thuricin 17 under optimal and salt stress conditions (n = 10); Means with the same letter are not significantly different (P>0.05). SEM = standard error of mean.

| Treatments | 24h | ±SEM | 30h | ±SEM | 36h | ±SEM | 48h | ±SEM |
| --- | --- | --- | --- | --- | --- | --- | --- | --- |
| Control (Water) | 62.00**^a^** | 6.03 | 93.00**^a^** | 4.28 | 96.00**^a^** | 2.59 | 99.00**^a^** | 1.50 |
| LCOA | 58.00**^a^** | 3.22 | 89.50**^a^** | 3.22 | 94.00**^a^** | 2.37 | 98.50**^b^** | 1.26 |
| LCOB | 60.00**^a^** | 3.54 | 91.25**^a^** | 3.54 | 95.00**^a^** | 2.25 | 98.75**^a^** | 1.37 |
| THA | 60.00**^a^** | 3.95 | 91.25**^a^** | 3.95 | 95.00**^a^** | 2.60 | 98.75**^ab^** | 1.51 |
| THB | 60.00**^a^** | 4.75 | 91.25**^a^** | 2.28 | 95.00**^a^** | 1.67 | 98.75**^ab^** | 0.37 |
|  |  |  |  |  |  |  |  |  |
| Control (100 mM NaCl) | 20.00**^b^** | 4.22 | 37.00**^b^** | 5.78 | 58.00**^b^** | 7.42 | 81.00**^b^** | 5.67 |
| LCOA + 100 mM NaCl | 22.22**^ab^** | 2.48 | 51.44**^ab^** | 4.23 | 83.00**^a^** | 4.96 | 94.00**^a^** | 3.06 |
| LCOB +100 mM NaCl | 32.00**^a^** | 3.89 | 60.00**^a^** | 4.47 | 80.00**^a^** | 4.94 | 93.00**^a^** | 3.00 |
| THA + 100 mM NaCl | 17.00**^b^** | 3.67 | 47.00**^ab^** | 7.61 | 71.00**^ab^** | 6.40 | 93.00**^a^** | 4.23 |
| THB + 100 mM NaCl | 25.00**^ab^** | 3.73 | 56.00**^a^** | 4.00 | 71.00**^ab^** | 3.48 | 92.00**^ab^** | 2.91 |
|  |  |  |  |  |  |  |  |  |
| Control (125 mM NaCl) | 1.00**^a^** | 1.00 | 17.00**^b^** | 2.60 | 30.00**^b^** | 3.94 | 76.00**^a^** | 4.27 |
| LCOA + 125 mM NaCl | 3.00**^a^** | 1.53 | 36.00**^a^** | 4.76 | 58.00**^a^** | 6.46 | 82.00**^a^** | 3.89 |
| LCOB +125 mM NaCl | 2.00**^a^** | 1.33 | 37.00**^a^** | 5.17 | 55.00**^a^** | 6.54 | 83.00**^a^** | 3.96 |
| THA + 125 mM NaCl | 4.00**^a^** | 1.63 | 32.00**^a^** | 4.67 | 53.00**^a^** | 5.97 | 84.00**^a^** | 3.71 |
| THB + 125 mM NaCl | 2.00**^a^** | 1.33 | 27.00**^ab^** | 4.73 | 49.00**^a^** | 7.67 | 81.00**^a^** | 4.33 |
|  |  |  |  |  |  |  |  |  |
| Control (150 mM NaCl) | 0.00**^a^** | 0.00 | 14.00**^b^** | 4.00 | 27.00**^c^** | 5.17 | 88.00**^a^** | 2.49 |
| LCOA + 150 mM NaCl | 0.00**^a^** | 0.00 | 17.00**^ab^** | 2.13 | 41.00**^ab^** | 3.79 | 79.00**^bc^** | 3.79 |
| LCOB +150 mM NaCl | 4.00**^a^** | 2.21 | 25.00**^a^** | 2.24 | 51.00**^a^** | 4.07 | 87.00**^ab^** | 3.67 |
| THA + 150 mM NaCl | 3.00**^a^** | 2.13 | 24.00**^a^** | 4.00 | 43.00**^ab^** | 6.16 | 80.00**^abc^** | 3.33 |
| THB + 150 mM NaCl | 1.00**^a^** | 1.00 | 16.00**^ab^** | 3.06 | 34.00**^bc^** | 3.40 | 73.00**^c^** | 2.13 |
|  |  |  |  |  |  |  |  |  |
| Control (175 mM NaCl) | 0.00**^a^** | 0.00 | 11.00**^a^** | 3.14 | 34.00**^bc^** | 4.00 | 83.00**^ab^** | 3.00 |
| LCOA + 175 mM NaCl | 1.00**^a^** | 1.00 | 17.00**^a^** | 3.96 | 44.00**^abc^** | 3.71 | 78.00**^b^** | 4.67 |
| LCOB +175 mM NaCl | 2.00**^a^** | 2.00 | 17.00**^a^** | 4.48 | 45.00**^ab^** | 5.43 | 90.00**^a^** | 3.65 |
| THA + 175 mM NaCl | 0.00**^a^** | 0.00 | 12.00**^a^** | 3.59 | 31.00**^c^** | 5.47 | 77.00**^b^** | 4.73 |
| THB + 175 mM NaCl | 0.00**^a^** | 0.00 | 20.00**^a^** | 3.33 | 49.00**^a^** | 4.33 | 84.00**^ab^** | 3.06 |
|  |  |  |  |  |  |  |  |  |
| Control (200 mM NaCl) | 1.00**^a^** | 1.00 | 8.00**^a^** | 2.00 | 23.00**^a^** | 3.35 | 66.00**^a^** | 5.81 |
| LCOA + 200 mM NaCl | 0.00**^a^** | 0.00 | 6.00**^a^** | 4.00 | 17.00**^ab^** | 4.48 | 66.00**^a^** | 4.76 |
| LCOB +200 mM NaCl | 0.00**^a^** | 0.00 | 2.00**^a^** | 1.33 | 10.00**^b^** | 2.98 | 67.00**^a^** | 4.73 |
| THA + 200 mM NaCl | 0.00**^a^** | 0.00 | 3.00**^a^** | 2.13 | 15.00**^ab^** | 3.73 | 69.00**^a^** | 2.77 |
| THB + 200 mM NaCl | 0.00**^a^** | 0.00 | 3.00**^a^** | 2.13 | 18.00**^ab^** | 4.16 | 72.00**^a^** | 4.90 |
