# Supplementary material for: A Proteomic Approach to Lipo-Chitooligosaccharide and Thuricin 17 Effects on Soybean GerminationUnstressed and Salt Stress
Source: PLoS One. 2016 Aug 25;11(8):e0160660. doi: 10.1371/journal.pone.0160660 (PMC4999219; doi:10.1371/journal.pone.0160660)
Supplement: S2 Table — (DOCX) [file pone.0160660.s004.docx]

S2 Table: Least square means of elemental analysis data on 48h post germinated soybean (Treatments include lipo-chito-oligosaccharide and thuricin 17 under optimal and salt stress conditions (n = 10)); Means with the same letter are not significantly different (P>0.05). SEM = standard error of mean.

| Treatments | %N | ± SEM | %C | ± SEM | N:C | ± SEM |
| --- | --- | --- | --- | --- | --- | --- |
|  |  |  |  |  |  |  |
| Control (Water) | 6.1549**^a^** | 0.0892 | 50.3368**^a^** | 0.3303 | 0.0445**^b^** | 0.0006 |
| LCOA | 6.2832**^a^** | 0.0291 | 50.5029**^a^** | 0.2075 | 0.0450**^b^** | 0.0003 |
| LCOB | 6.3782**^a^** | 0.2540 | 51.9692**^a^** | 2.2805 | 0.0452**^b^** | 0.0004 |
| THA | 6.2687**^a^** | 0.0515 | 50.5091**^a^** | 0.2383 | 0.0455**^b^** | 0.0004 |
| THB | 6.2722**^a^** | 0.0576 | 50.0379**^a^** | 0.4552 | 0.0475**^a^** | 0.0010 |
|  |  |  |  |  |  |  |
| Control (100 mM NaCl) | 6.2045**^a^** | 0.0693 | 49.0413**^a^** | 0.1073 | 0.0518**^a^** | 0.0005 |
| LCOA + 100 mM NaCl | 6.3118**^a^** | 0.0403 | 49.1772**^a^** | 0.0964 | 0.0523**^a^** | 0.0003 |
| LCOB +100 mM NaCl | 6.2174**^a^** | 0.0670 | 49.0952**^a^** | 0.0593 | 0.0519**^a^** | 0.0006 |
| THA + 100 mM NaCl | 6.2496**^a^** | 0.0848 | 48.6358**^b^** | 0.2460 | 0.0525**^a^** | 0.0009 |
| THB + 100 mM NaCl | 6.2581**^a^** | 0.0502 | 49.0804**^a^** | 0.1198 | 0.0517**^a^** | 0.0002 |
|  |  |  |  |  |  |  |
| Control (125 mM NaCl) | 6.0832**^ab^** | 0.0298 | 48.2801**^bc^** | 0.2101 | 0.0487**^a^** | 0.0009 |
| LCOA + 125 mM NaCl | 6.0553**^b^** | 0.0461 | 47.9025**^c^** | 0.1850 | 0.0491**^a^** | 0.0009 |
| LCOB +125 mM NaCl | 6.1021**^ab^** | 0.0361 | 48.3505**^bc^** | 0.1726 | 0.0487**^a^** | 0.0007 |
| THA + 125 mM NaCl | 6.1474**^ab^** | 0.0275 | 48.5420**^b^** | 0.1362 | 0.0464**^b^** | 0.0004 |
| THB + 125 mM NaCl | 6.1735**^a^** | 0.0256 | 49.0900**^a^** | 0.1181 | 0.0457**^b^** | 0.0004 |
|  |  |  |  |  |  |  |
| Control (150 mM NaCl) | 6.0127**^a^** | 0.0451 | 48.7479**^c^** | 0.1690 | 0.0496**^b^** | 0.0004 |
| LCOA + 150 mM NaCl | 6.0800**^a^** | 0.0347 | 49.2309**^ab^** | 0.1109 | 0.0503**^ab^** | 0.0003 |
| LCOB +150 mM NaCl | 6.0098**^a^** | 0.0546 | 49.6169**^a^** | 0.1627 | 0.0495**^b^** | 0.0004 |
| THA + 150 mM NaCl | 6.1066**^a^** | 0.0820 | 48.9219**^bc^** | 0.1634 | 0.0513**^a^** | 0.0008 |
| THB + 150 mM NaCl | 6.0191**^a^** | 0.0475 | 49.1470**^bc^** | 0.1098 | 0.0501**^ab^** | 0.0005 |
|  |  |  |  |  |  |  |
| Control (175 mM NaCl) | 6.1830**^b^** | 0.0498 | 49.8454**^a^** | 0.2135 | 0.0510**^b^** | 0.0004 |
| LCOA + 175 mM NaCl | 6.2802**^ab^** | 0.0615 | 50.1444**^a^** | 0.0398 | 0.0515**^ab^** | 0.0006 |
| LCOB +175 mM NaCl | 6.3932**^a^** | 0.0913 | 51.0387**^a^** | 0.9265 | 0.0515**^ab^** | 0.0004 |
| THA + 175 mM NaCl | 6.3182**^ab^** | 0.0567 | 50.5682**^a^** | 0.1467 | 0.0511**^b^** | 0.0005 |
| THB + 175 mM NaCl | 6.4595**^a^** | 0.0661 | 50.3934**^a^** | 0.1118 | 0.0525**^a^** | 0.0005 |
|  |  |  |  |  |  |  |
| Control (200 mM NaCl) | 6.3023**^a^** | 0.0358 | 49.5587**^a^** | 0.1302 | 0.0522**^ab^** | 0.0003 |
| LCOA + 200 mM NaCl | 6.2913**^a^** | 0.0418 | 49.7222**^a^** | 0.1111 | 0.0524**^a^** | 0.0004 |
| LCOB +200 mM NaCl | 6.2438**^ab^** | 0.0448 | 49.4051**^a^** | 0.1484 | 0.0522**^ab^** | 0.0004 |
| THA + 200 mM NaCl | 6.2311**^ab^** | 0.0441 | 49.5906**^a^** | 0.3052 | 0.0515**^ab^** | 0.0003 |
| THB + 200 mM NaCl | 6.1246**^b^** | 0.0600 | 49.3233**^a^** | 0.1857 | 0.0511**^b^** | 0.0006 |
