# Supplementary material for: A Proteomic Approach to Lipo-Chitooligosaccharide and Thuricin 17 Effects on Soybean GerminationUnstressed and Salt Stress
Source: PLoS One. 2016 Aug 25;11(8):e0160660. doi: 10.1371/journal.pone.0160660 (PMC4999219; doi:10.1371/journal.pone.0160660)
Supplement: S3 Table — (DOCX) [file pone.0160660.s005.docx]

S3 Table: GO function categories amongst un-stressed and salt stressed groups

| Molecular function | No. of seq  Signals | No. of seq  Salt | Biological process | No. of seq  Signals | No. of seq  Salt | Cellular component | No. of seq  Signals | No of seq Salt |
| --- | --- | --- | --- | --- | --- | --- | --- | --- |
| Structural constituent of ribosome | 356 | 300 | Response to cadmium ion | 384 | 294 | Plasma membrane | 721 | 584 |
| ATP binding | 267 | 170 | Translation | 343 | 288 | Cholorplast | 394 | 344 |
| Nucleotide binding | 115 | 390 | **Oxidation-reduction process** | **280** | **0** | Cytosol | 362 | 242 |
| Protein binding | 105 | 115 | Response to salt stress | 260 | 175 | Nucleus | 340 | 273 |
| Nutrient reservoir activity | 101 | 85 | Glycolysis | 132 | 61 | Vacuole | 295 | 279 |
| ATPase activity | **93** | **0** | Gluconeogenesis | 128 | 78 | Nucleolus | 293 | 235 |
| Copper ion binding | 84 | 82 | Response to misfold protein | 120 | 50 | Cytosolic ribosome | 197 | 170 |
| *GTP binding* | ***83*** | ***83*** | Toxin catabolic process | 118 | 45 | Apoplast | 187 | 159 |
| Calcium ion binding | 83 | 77 | Proteasomal ubiquitin-dependent protein catabolic process | 117 | 50 | Cell wall | 184 | 142 |
| Metal ion binding | 80 | 40 | Fatty acid beta oxidation | 116 | 45 | ***Cytoplasm*** | ***154*** | ***152*** |
| 2-alkenal reductase [(NAD)P] activity | 78 | 62 | **Proteasome core complex** | **112** | **0** | ***Mitochondrion*** | ***149*** | ***147*** |
| Peptidase activity | 66 | 67 | Response to cold | 96 | 78 | Chloroplast stroma | 134 | 118 |
| DNA binding | 65 | 64 | Response to heat | 95 | 51 | Chloroplast envelope | 125 | 108 |
| *Protein heterodimerization activity* | ***60*** | ***60*** | **Photorespiration** | **87** | **0** | **Proteasome regulatory particle, base subcomplex** | **95** | **0** |
| GTPase activity | 59 | 62 | ***Protein folding*** | ***79*** | ***79*** | Ribosome | 90 | 81 |
| Heme binding | **58** | **0** | Response to stress | 78 | 48 | Membrane | 88 | 78 |
| RNA binding | 55 | 48 | **Meristem structural organization** | **72** | **0** | Plant-type cell wall | 84 | 82 |
| Zinc ion binding | 53 | 51 | **Response to oxidative stress** | **68** | **0** | Cytosolic large ribosome | 83 | 77 |
| *Translation elongation factor activity* | ***46*** | ***49*** | Proteolysis | 67 | 70 | Integral to membrane | 79 | 63 |
| Unfolded protein binding | 42 | 39 | **Leaf morphogenesis** | **64** | **0** | Small ribosomal subunit | 78 | 71 |
| Nucleotidyltransferases activity | **0** | **34** | **Nucleosome assembly** | **0** | **64** | **Nucleosome** | **0** | **60** |
| Cation binding | **0** | **36** | **GTP catabolic process** | **0** | **61** |  |  |  |
|  |  |  | **Cytoskeletol organization** | **0** | **55** |  |  |  |
|  |  |  | **Mature ribosome assembly** | **0** | **52** |  |  |  |
